# P-115. Lessons from Establishing a Vaccine Trial Clinic: Insights from the University of Louisville's Center of Excellence for Research in Infectious Diseases

**DOI:** 10.1093/ofid/ofae631.322

**Published:** 2025-01-29

**Authors:** Nouman Shafique, Waqas Shahnawaz, Dawn Balcom

**Affiliations:** University of Louisville, Louisville, Kentucky; University of Louisville, Louisville, Kentucky; University of Louisville, Louisville, Kentucky

## Abstract

**Background:**

The COVID-19 pandemic spurred an urgent need for swift vaccine development to combat the disease. This study focuses on outlining the implementation approach for the Phase 3 clinical trial of the Ad26.COV2.S vaccine. The University of Louisville's Division of Infectious Diseases, known as the Center of Excellence for Research in Infectious Diseases (CERID), was contacted by Janssen representatives in July 2020 to conduct the trial.

**Figure 1.**
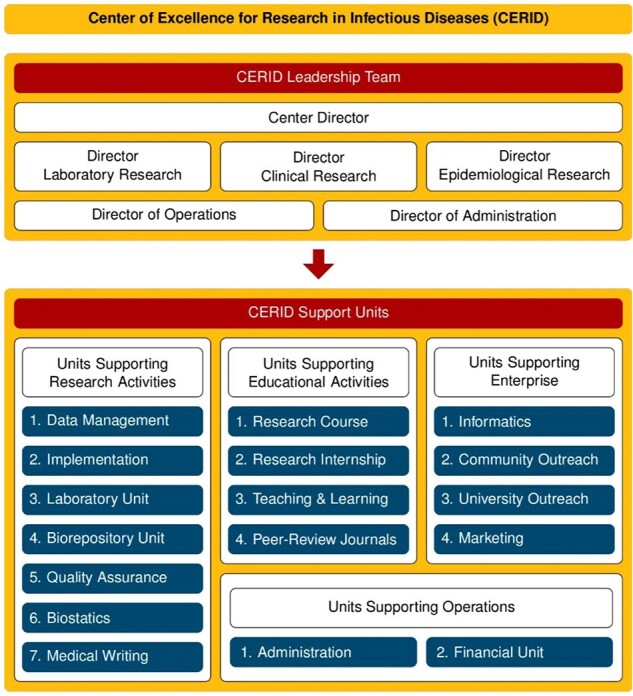
The structure of the Center of Excellence for Research in Infectious Diseases (CERID).

**Methods:**

The CERID site was fully activated in November 2020, and the enrollment period spanned 16 working days, from November 21 to December 11, 2020. The study employed a randomized, double-blind, placebo-controlled design. Various organizational units within CERID, including community outreach, implementation, quality assurance, and laboratory, collaborated to develop standard operating protocols for communication and execution. These steps included community outreach, trainings, and the development of case report forms.

**Figure 2.**
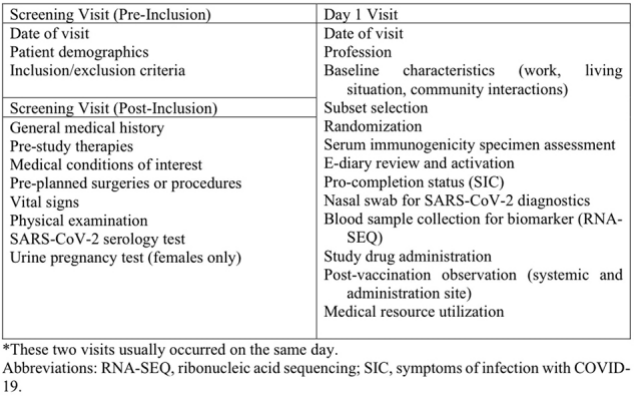
Data collected at Screening and Day 1 participant visits.

**Results:**

During the enrollment period, 64 participants were seen, with 62 enrolled in the trial. The self-sufficient organizational infrastructure of CERID was pivotal in facilitating the successful implementation of the Phase 3 clinical trial. Quality metrics were developed to minimize systematic errors and ensure accurate reporting of results. The Division of Infectious Diseases Clinical Laboratory Improvement Amendments (CLIA)-certified laboratory provided essential support for COVID-19 diagnosis, sample processing, and shipping.

**Figure 3.**
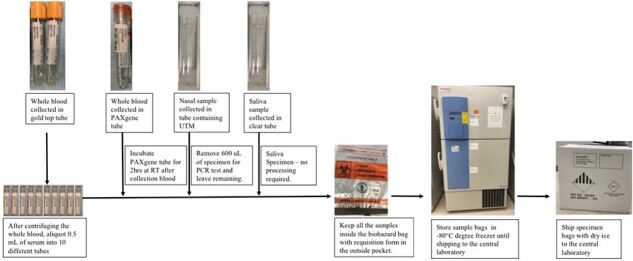
Workflow in laboratory. Gold top humoral blood collection tube, PAXgene RNA blood sequencing tube, NP specimen tube, and saliva samples were delivered to the laboratory. Specimens processed per laboratory manual and stored in the -80°C freezer until shipping to the central laboratory.

**Conclusion:**

The seamless execution of the Phase 3 clinical trial at CERID highlights the effectiveness of a multidisciplinary research coordinating center. The collaborative efforts of various units within CERID, along with proactive measures such as pre-labeling specimen tubes and addressing participant concerns directly, contributed to the trial's success. The described methods and approach can be adapted for use in diverse clinical and laboratory research settings, showcasing resilience and adaptability in the face of pandemic challenges

**Disclosures:**

**All Authors**: No reported disclosures

